# Enuresis and Hyperactivity-Inattention in Early Adolescence: Findings from a Population-Based Survey in Tokyo (Tokyo Early Adolescence Survey)

**DOI:** 10.1371/journal.pone.0158786

**Published:** 2016-07-14

**Authors:** Sho Kanata, Shinsuke Koike, Shuntaro Ando, Atsushi Nishida, Satoshi Usami, Syudo Yamasaki, Yuko Morimoto, Rie Toriyama, Shinya Fujikawa, Noriko Sugimoto, Tsukasa Sasaki, Toshiaki A. Furukawa, Mariko Hiraiwa-Hasegawa, Kiyoto Kasai

**Affiliations:** 1 Department of Neuropsychiatry, Graduate School of Medicine, The University of Tokyo, Tokyo, Japan; 2 Department of Psychiatry, Teikyo University School of Medicine, Tokyo, Japan; 3 University of Tokyo Institute for Diversity and Adaptation of Human Mind (UTIDAHM), The University of Tokyo, Tokyo, Japan; 4 Center for Evolutionary Cognitive Sciences, Graduate School of Art and Sciences, The University of Tokyo, Tokyo, Japan; 5 Department of Psychiatry and Behavioral Sciences, Tokyo Metropolitan Institute of Medical Science, Tokyo, Japan; 6 Faculty of Human Sciences, Division of Psychology, University of Tsukuba, Tsukuba, Japan; 7 Department of Evolutionary Studies of Biosystems, School of Advanced Sciences, SOKENDAI (The Graduate University for Advanced Studies), Hayama, Japan; 8 Laboratory of Health Education, Graduate School of Education, The University of Tokyo, Tokyo, Japan; 9 Department of Health Promotion and Human Behavior, Kyoto University Graduate School of Medicine / School of Public Health, Kyoto University, Kyoto, Japan; IRCCS E. Medea, ITALY

## Abstract

**Background:**

Enuresis (9% at age 9.5) negatively affects children’s psychosocial status. Clinically-diagnosed enuresis (2% at the age) is associated with hyperactivity-inattention, and common neural bases have been postulated to underlie this association. It is, however, unclear whether this association is applicable to enuresis overall among the general population of early adolescents when considered comorbid behavioral problems. We aimed to examine whether enuresis correlates with hyperactivity-inattention after controlling for the effects of other behavioral problems.

**Methods:**

Participants were 4,478 children (mean age 10.2 ± 0.3 years old) and their parents from the Tokyo Early Adolescence Survey (T-EAS), a population-representative cross-sectional study conducted in Tokyo, Japan conducted from 2012 to 2015. Children’s enuresis and behavioral problems, including hyperactivity-inattention (as measured by the Strength and Difficulties Questionnaire), were examined using parent-reporting questionnaires. Multivariate linear regression was used to explore whether enuresis predicts hyperactivity-inattention.

**Results:**

The hyperactivity-inattention score was significantly higher in the enuretic group than the non-enuretic group (enuretic: *M* (*SD*) = 3.8 (2.3), non-enuretic: *M* (*SD*) = 3.0 (2.1), Hedge’s *g* = 0.39, *p* < .001). This association remained significant even after controlling for other behavioral problems and including sex, age, intelligence quotient (IQ), low birth weight and parents’ education (β = .054 [95% CI: .028–.080], *p* < .001).

**Conclusions:**

Enuresis was independently associated with hyperactivity-inattention in early adolescents among general population even when other behavioral problems were considered. These results suggest that, as with clinically-diagnosed cases, enuresis may predict need for screening and psychosocial support for hyperactivity-inattention.

## Introduction

Enuresis, or pathological bedwetting, is defined as an intermittent (i.e., not continuous) wetting with any frequency during sleep in children more than or equal to five years old, according to the International Children’s Continence Society (ICCS) [1]. Epidemiological studies have shown that enuresis affects 25–30% of children at age 5 years, and 9.4% at 9.5 years [2,3]; whereas 5% of children at age 5 years and 2% at 10 [4] according to the Diagnostic and Statistical Manual of Mental Disorders (DSM-IV/5), which requires twice or more in any one week [5,6]. Although the biological basis of enuresis is not fully understood, clinical studies have postulated that there exists neurodevelopmental immaturity, especially in the brainstem and motor cortex circuitry [7,8], as well as urological deficits [9].

Enuresis, especially in clinical situation, is known to be associated with a range of children’s behavioral problems such as emotional problems, peer-relational problems, and conduct problems [10–12]. Hyperactivity-inattention, including attention-deficit hyperactivity disorder (ADHD), which is a pathologic condition on a hyperactivity-inattention spectrum [13], is the most common problem associated with enuresis, a link reported not only in clinical [14, 15], but also in epidemiological studies [16, 17]. A common neural basis is thought to underlie this association, perhaps through inhibitory regulation subserved by the anterior cingulate and the prefrontal cortexes [18], which develops through childhood and adolescence and matures in early adulthood [19, 20]. In clinical situations, these findings have been used for effective treatment of enuresis; the ICCS recommends screening enuretic children for hyperactivity-inattention, since simultaneous treatment for both leads to better outcomes in enuresis [10].

Children in early adolescence, at around 10 years old, perceive enuresis as a highly distressing problem, ranking it eighth among stressful life events [21], and children in eight years of age or older with persistent enuresis have psychosocial difficulties such as decreased self-esteem [22]. Appropriate care for enuresis is therefore very important, even if it does not appear in clinical situations or health professionals have not yet intervened. It is not, however, known whether hyperactivity-inattention may be independently associated with enuresis among general population as well as clinically-diagnosed enuresis [15]. Previous studies on the association have mainly been done in clinical situations, but low consulting rate for medical clinicians for enuresis [23] and restrictive criteria about frequency (twice or more per week according to the DSM-IV/5) [24] have excluded a considerable number of enuretic children from the studies [25].

A few epidemiological studies on early adolescents have suggested an association between enuresis and hyperactivity-inattention among general population. Byrd *et al*. reported that children with any episodes of enuresis in the previous year showed higher extreme behavior scores, including hyperactivity-inattention, than non-enuretic children [3]. Joinson *et al*. reported that current enuresis was associated with increased hyperactivity-inattention even after children with frequent enuresis such as twice or more per week were excluded from the analyses [25]. These studies did not, however, examine and separate the overlap of behavioral problems despite their high comorbidity among enuretic children [26].

It therefore remains unclear whether there is an independent association between enuresis and hyperactivity-inattention among general population when other behavioral problems such as emotional and conduct problems are also considered [25]. Our study aimed to examine whether enuresis is independently associated with hyperactivity-inattention in early adolescence even after controlling for the effects of other behavioral problems.

## Materials and Methods

### Study procedures and participants

This study used data from Tokyo Early Adolescence Survey (T-EAS), a population-based cross-sectional survey focusing on children’s health from bio-psycho-social multidisciplinary viewpoints. The T-EAS was also intended to serve as a baseline survey for a longitudinal study, which is now under way (Tokyo TEEN Cohort Study; TTC, http://ttcp.umin.jp/). The TTC, especially focusing on children’s self-regulation, aims to investigate development of children’s mental health throughout adolescence, which serves as a foundation for their future health [27]. The T-EAS was conducted from October 2012 to January 2015, and the participants were recruited from three municipalities in Tokyo (Setagaya, Mitaka, and Chofu) using the Basic Resident Register. Out of 18,830 children born between 1st September 2002 and 31st August 2004 and living in the municipalities at the time of the survey, 14,553 children, together with their parents, were randomly selected and invited to participate in the survey. We were unable to contact 4,319 of these 14,553 child/parent groups. Of the 10,234 child/parent groups who were contacted, 5,756 refused to participate, leaving a total of 4,478 participating children, together with their parents (a response rate of 43.8%).

Using a parent-reported questionnaire children were excluded from the analyses if they needed care for urological and neurological deficits other than enuresis, or if they had taken medication for these conditions in the previous 2 weeks (*n* = 44). Of the 4,434 participants, data from 90 participants were not used for the final regression analysis owing to missing values, leaving complete data from 4,344 child/parent groups available for the final analyses. There was no significant difference in demographic characteristics between those included and not included in the final analyses (*p* > .10).

### Measures

Specially trained interviewers visited participants’ homes and collected data. They obtained informed consent for participation, distributed questionnaires to the children and parents, and conducted psychological tests for the children.

#### Enuresis

Parents were asked about their children’s enuresis using the question “Does your child currently wet the bed at night?” with the following options: (1) Never, (2) Sometimes, (3) Once or twice a week, (4) Three or four times a week, and (5) Using diapers. The answers were converted to a dichotomous variable, and children with any enuresis (answers (2)–(5)) were defined as an enuretic group with the others (an answer (1)) defined as a non-enuretic group.

Children who experienced enuresis frequent more than once a week (answers (3)–(5)), or had a diagnosis and medication for enuresis were regarded as a frequent enuresis group.

#### Children’s behavioral problems

We employed the Japanese version of the Strength and Difficulties Questionnaire (SDQ) [28], a widely-used parent-reported questionnaire developed to evaluate behavioral conditions of children and adolescents [29]. The SDQ has four difficulty subscales (hyperactivity-inattention, emotional symptoms, conduct problems, and peer-relationship problems), and each of the subscales has five questions with 3-point Likert scales (0: Not true, 1: Somewhat true, 2: Absolutely true). The scores are summed for each subscale, so that each has a possible score from 0 to 10.

#### Other variables

Sex, age, intelligence quotient (IQ), low birth weight, and parents’ educational backgrounds were included in the analyses since they are known to relate to children’s enuresis and behavioral problems [4,28,30–32]. Children’s IQ was estimated by a short version of the Wechsler Intelligence Scale for Children (WISC-III), which consists of two subsets (Information and Picture Completion) [33]. We developed a formula (Estimated IQ = 3.3*Information + 2.0*Picture Completion + 45.6) to estimate children’s IQ from the two subsets using data from 28 children who were tested using the full version of WISC-III by expert psychologists one year after participation in T-EAS. The IQ calculated by the formula explained 78% of the variance in IQ from the full version of WISC-III. Birth weight was collected from the Maternal and Child Health Handbook, which are distributed to all pregnant women by the local government in Japan to record maternal and child medical and health information during pregnancy and after birth. We used a dichotomous variable for low birth weight (< 2,500 g), in line with the tenth version of the International Classification of Diseases [34]. To assess parents’ educational backgrounds, we asked about their highest level of education using six items [35]: (1) Junior high school (or less), (2) High school (leaving before graduation), (3) High school (graduated), (4) Vocational school or two-year college, (5) Four-year college or university, and (6) Postgraduate education (or more).

### Statistical analysis

We examined differences between the enuretic and non-enuretic groups in the SDQ subscales and other variables using *t*-tests, and calculated the effect sizes using Hedge’s *g*. We then carried out multivariate linear regression analyses to examine whether enuresis is independently associated with hyperactivity-inattention; the independent variable was enuresis, and the dependent variable was the SDQ subscale score for hyperactivity-inattention. The analyses had two models. Model 1 included potential confounding variables such as sex, age, estimated IQ, low birth weight, and parents’ educational backgrounds, which were forced into the model simultaneously. Model 2 added the other SDQ subscales as well as the confounders in model 1. To assess the independent association between enuresis and the other SDQ subscales, we repeated the same analyses for each of the other SDQ subscales. As previous studies have shown sex differences in prevalence both of enuresis and behavioral problems [3, 4, 28], the effect of interactions between sex and enuresis on each subscale of the SDQ were also evaluated in both models. Finally, as sensitivity analyses, the statistical procedures above were repeated after excluding the frequent enuresis group.

The threshold for statistical significance was set to *p* < .05 (two-sided) for all analyses. All statistical analyses were carried out using SPSS (Statistical Package for Social Science; IBM Corp., Armonk, NY USA), version 21.0 for Macintosh.

### Ethical approval

Ethical approval for this study was obtained from the research ethics committees of Tokyo Metropolitan Institute of Medical Science (Approval number: 12–35), SOKENDAI (The Graduate University for Advanced Studies) (2012002), and the Graduate School of Medicine and Faculty of Medicine, The University of Tokyo (10057). Informed consent was obtained from both children and parents and written informed consent was obtained from the parents.

## Results

### Prevalence of enuresis

Enuresis was reported for 407 (9.2%) children out of 4,434 participants (Table 1). Of these, 61 children (1.4% of the total) were placed in the frequent enuresis group. Boys were more likely to show both enuresis (OR = 2.66 [95% CI: 2.12–3.33], *p* < .001), and frequent enuresis (OR = 2.30 [95% CI: 1.31–4.05], *p* = .0036).

**Table 1:** Demographic data of the participants (Total *N* = 4,434).

Variables	*n* / Mean	(%) / *SD*
**Sex, male**	2347	(52.9)
**Age**	10.2	0.3
**Estimated IQ**	107.8	14.0
**Birth Weight**, g	3025	412
Low Birth Weight (< 2,500 g)	354	(8.1)
**Father’s education**		
Junior high school or less	16	(0.4)
High school (leaving before graduation)	90	(2.1)
High school (graduated)	606	(14.3)
Vocational school or two-year college	574	(13.6)
Four-year college or university	2419	(57.2)
Postgraduate education or more	527	(12.5)
**Mother’s education**		
Junior high school or less	11	(0.3)
High school (leaving before graduation)	41	(0.9)
High school (graduated)	678	(15.4)
Vocational school or two-year college	1909	(43.4)
Four-year college or university	1614	(36.7)
Postgraduate education or more	141	(3.2)
**Enuresis**		
None	4014	(90.8)
Sometimes	353	(8.0)
Once or twice a week	20	(0.5)
Three or four times a week	7	(0.2)
Using diapers	27	(0.6)
**Strength Difficulties Questionnaire**		
Hyperactivity-inattention	3.03	2.14
Emotional symptoms	1.57	1.69
Conduct problems	1.84	1.53
Peer relationship problems	1.52	1.57

Abbreviations: *SD*, Standard Deviation; IQ, Intelligence quotient.

### Differences in behavioral problems and other variables between the enuretic and non-enuretic groups

The enuretic group had a significantly higher hyperactivity-inattention score than the non-enuretic group (enuretic: mean (*SD*) = 3.8 (2.3), non-enuretic: 3.0 (2.1), *p* < .001, Table 2). This difference corresponded with Hedge’s *g* = 0.39. The other SDQ subscale scores were also higher among the enuretic group (*g* = 0.18 *vs*. 0.26, *p* < .001). There were no significant differences in children’s age, estimated IQ, and parental education (*p* > .20) between the two groups. These results did not change significantly even after excluding the frequent enuresis group (hyperactivity-inattention, enuretic: mean (*SD*) = 3.8 (2.3), non-enuretic: 3.0 (2.1), *g* = 0.38, *p* < .001).

**Table 2:** Comparison of behavioral problems in the enuretic and non-enuretic groups.

	Non-enuretic		Enuretic			*p*-value	Effect size
	(*n* = 3997)		(*n* = 407)		*t*		
	Mean	*SD*	Mean	*SD*			
SDQ							
Hyperactivity-inattention	2.96	2.11	3.79	2.26	7.11	< .001***	**0.39**
Emotional symptoms	1.55	1.67	1.84	1.85	3.10	< .001***	**0.18**
Conduct problems	1.80	1.51	2.18	1.66	4.43	< .001***	**0.25**
Peer relationship problems	1.49	1.55	1.81	1.74	3.51	< .001***	**0.20**

Effect size was Hedge’s *g*. Statistical significances were tested by *t*-test for two means.

Abbreviations: *SD*, Standard Deviation; SDQ, the Strength and Difficulties Questionnaire

*** *p* < .001

### Independent association between enuresis and behavioral problems

Enuresis was associated with increased scores in hyperactivity-inattention and all the other SDQ subscales after controlling for demographic variables (hyperactivity-inattention: β = .087 [95% CI:.057–.117], *p* < .001, other subscales: β = .048–.059, *p* < .005, Table 3, model 1). In model 2, which additionally adjusted for the other SDQ subscales, enuresis was associated only with increased hyperactivity-inattention (β = .054 [95% CI: .028–.080], *p* < .001, model 2), but not with the other SDQ subscales (*p* > .15). Sensitivity analyses, which excluded the frequent enuresis group, showed similar results in both models (hyperactivity-inattention: β = .049 [95% CI: .023–.076], *p* < .001, other subscales: *p* > .15, model 2).

**Table 3:** Multivariate linear regression analyses predicting behavioral problems from enuresis.

	Model 1 ^a^^)^			Model 2 ^b^^)^		
	β	95% C.I.	*p*-value	β	95% C.I.	*p*-value
SDQ						
Hyperactivity inattention	**.087**	**[.057–.117]**	< .001***	**.054**	**[.028–.080]**	< .001***
Emotional symptoms	**.052**	**[.022–.083]**	< .001***	.020	[−.008–.048]	.16
Conduct problems	**.059**	**[.029–.090]**	< .001***	.012	[−.015–.039]	.37
Peer relationship problems	**.048**	**[.017–.079]**	.0022**	.015	[−.014–.043]	.31

Final number included in analyses *n* = 4,344. Dependent variable was each SDQ subscale, and independent variable was enuresis. All the variables were forced into the model simultaneously, and multi-collinearity was not indicated (*VIF* < 1.5).

a) Adjusted for age, sex, estimated IQ, father’s education, mother’s education, low birth weight.

b) Adjusted for all of the other subscales of the SDQ as well as those in model 1.

Abbreviations: SDQ Strength and Difficulties Questionnaire; IQ Intelligence quotient; C.I. Confidence Interval

** *p* < .01,

*** *p* < .001.

There were no significant interaction effects between enuresis and sex on the SDQ subscales in these multivariate linear regression analyses (*p* > .20). Interaction terms were therefore removed from the final analyses shown in Table 3.

## Discussion

This study showed that enuresis was independently associated with hyperactivity-inattention in the general population of early adolescents even when considering other behavioral problems such as emotional symptoms, conduct problems, and peer-relationship problems. These results were essentially unchanged even after the frequent enuresis group was excluded.

To the best of our knowledge, this is the first study that has showed the independent association between enuresis and hyperactivity-inattention among a large general population in early adolescence. The result was consistent with a previous case-control clinical study showing independent association of hyperactivity-inattention with enuresis [15]. Our results could have adequate reliability and validity since the prevalence and sex ratio of enuresis is consistent with previous studies [3, 4]. We also observed significant associations between enuresis and other behavioral problems such as emotional and conduct problems in the unadjusted model (model 1), in line with a previous epidemiological study [25]. The sensitivity analyses excluding the frequent enuresis group did not significantly change the results. Therefore, enuresis may not be clearly divided into pathologic/non-pathologic groups according to its frequency, although the DSM-IV/5 defines enuresis based on the frequency [24].

No independent association with enuresis was observed for the other behavioral problems in this study. The clinical case-control study cited above, however, reported that not only hyperactivity-inattention but all the other behavioral problems in the SDQ were also independently associated with enuresis [15]. This difference in the results might be due to the small number of participants recruited from clinical patients in the previous study. Stronger distress associated with enuresis in clinical situation, rather than enuresis without consulting to clinicians, might lead to the association of enuresis with other behavioral problems as well as hyperactivity-inattention [36].

Although the mechanisms for the association between enuresis and hyperactivity-inattention remain unclear, we can propose some possible explanations based on previous studies. From a psychosocial viewpoint, children’s increased hyperactivity-inattention could be a relapse of coping behavior resulted from the psychological burdens of enuresis [9, 36]. These burdens become much stronger in early adolescence, from around 10 years, since enuresis increasingly begins to carry social and peer-relational meanings at that point [21, 37]. From a biological viewpoint, neurophysiological and neuroimaging studies have recently suggested common developmental immaturities in the central nervous system for both enuresis and hyperactivity-inattention [18, 38]. A neurophysiological case-control study showed that deficits in prepulse inhibition were greater in children with clinically-diagnosed enuresis or attention deficit hyperactivity disorder (ADHD) than healthy controls, and greatest in children with both conditions [39]. These results could imply that the two conditions share dysregulation of inhibitory signal processing system in the pons, where exists the pontine micturition center [40], modulated by limbic cortico-striato-pallido-thalamic circuitry and prefrontal neural circuitry [41]. There has been no neuroimaging study focusing on the direct association between enuresis and hyperactivity-inattention. Functional magnetic resonance imaging studies during Go/No-Go tasks, however, have shown impaired activities in the anterior cingulate and the prefrontal cortexes, which are responsible for self-regulatory behavior against emotional reward cues [18–20], in patients with both clinical enuresis [42] and ADHD [43].

The results of this study may provide some clues for appropriate care for children showing enuresis and not consulting to clinicians. In clinical situations, for effective treatment of enuresis, the ICCS recommends screening for psycho-behavioral problems, especially hyperactivity-inattention, among enuretic children and provision of psychosocial care for both issues [10]. This study’s findings suggest that this recommendation could also be applicable to enuresis in non-clinical situations, and that screening for hyperactivity-inattention and psychosocial care for the problem might prove effective care for enuretic children in early adolescence. This implication could be true of not only clinicians and parents but also school teachers and social welfare workers, who often face children with enuresis and hyperactivity-inattention in non-clinical situations (at home, school, and community).

This study has several limitations. First, our survey included no physical examination by medical specialists for urological and neurological disorders. Instead we relied on the parents’ reports. This might not have been sufficient, as physical examinations are recommended in studying incontinence problems [38]. This meant that we were unable to guarantee exclusion of children with urinary disorders other than enuresis. Second, the SDQ scores might be biased towards high scores in the enuretic group, since parents of enuretic children, who perceive higher stress than ones of non-enuretic children [12], could negatively affect their evaluation of children’s behavioral problems [44]. This limitation would, however, not explain the independent association of enuresis with hyperactivity-inattention, and not with other behavioral problems. Third, even though hyperactivity-inattention and ADHD are characterized as a continuous spectrum [13], it requires careful attention that children with high score of hyperactivity-inattention in the SDQ does not necessarily receive a diagnosis of ADHD. Finally, the cross-sectional nature of this study means that we cannot draw conclusions about any causal relationship between children’s enuresis and hyperactivity-inattention. Future studies would benefit from drawing on both prospective and longitudinal viewpoints to clarify the causality. We plan to investigate this in the current longitudinal study.

## Conclusion

Enuresis was associated with increased hyperactivity-inattention among the general population of early adolescents even after controlling for other behavioral problems. The lines of evidence on treatments for clinical enuresis, such as screening and psychosocial support for hyperactivity-inattention [10], might therefore also be applicable to early adolescents showing enuresis even if not appearing to clinical situations.
